# Cocaine as a rare cause of locked-in syndrome: a case report

**DOI:** 10.1186/s13256-019-2278-2

**Published:** 2019-11-19

**Authors:** Osman Ali, Mark G. Bueno, Trinh Duong-Pham, Nuwan Gunawardhana, Dena H. Tran, Robert D. Chow, Avelino C. Verceles

**Affiliations:** 1 Division of Pulmonary & Critical Care Medicine, University of Maryland School of Medicine, 110 S. Paca Street, Baltimore, MD 21201 USA; 2 Division of Internal Medicine, University of Maryland Midtown Campus, 827 Linden Avenue, Baltimore, MD 21201 USA; 3 Division of Pulmonary, Critical Care & Sleep Medicine, University of Maryland School of Medicine, 110 S. Paca Street, Baltimore, MD 21201 USA

**Keywords:** Basilar artery, Cocaine-related disorders, Magnetic resonance imaging, Quadriplegia, Locked-in syndrome

## Abstract

**Introduction:**

In the United States, cocaine is a commonly used drug of abuse. It is also a recognized contributing factor for both hemorrhagic and ischemic strokes. However, cocaine-induced basilar artery thrombosis has rarely been reported in the literature.

**Case presentation:**

Our patient was a 51-year-old African American woman with a history of polysubstance abuse who presented to the emergency department for acute behavior changes. Later, during admission, she had a dramatic decrease in motor strength in all extremities and a positive Babinski reflex bilaterally. The results of her toxicology reports were positive for cocaine; in addition, results of magnetic resonance angiography and magnetic resonance imaging were consistent with acute thrombosis and subsequent infarction of the basilar artery. Her mental status improved, but she was only able to communicate via movements of her eyes.

**Conclusion:**

Our patient developed locked-in syndrome after use of cocaine. Given the prevalence of its use in the United States, cocaine use should be included among the potential causes of locked-in syndrome.

## Introduction

According to the National Survey on Drug Use and Health (NSDUH), cocaine is the second most popular recreational drug in the United States behind marijuana. The NSDUH reported in 2008 that 36.7 million people in the United States had used cocaine during their lifetime [1]. In 2012, the NSDUH found that nearly 4.7 million Americans aged 12 or older reported using cocaine in the past year, and almost 38 million Americans reported ever using cocaine in their lifetime [2].

Locked-in syndrome (LIS) is a rare manifestation of stroke and is typically challenging for clinicians to diagnose, taking an average of 78 days to uncover [3]. The syndrome is characterized by the following findings: paralysis of the limbs and oral structures, cranial nerve deficits, and intact consciousness with retention of cognitive function and vertical eye movement [4]. The first contemporary description of the syndrome was published in 1941 by Cairns and associates [5]. In 1966, Plum and Posner coined the term *locked-in syndrome* in their description of a similar case [6]. LIS results from a lesion affecting the corticospinal, corticobulbar, and corticopontine tracts of the ventral pons, with the most common cause of LIS being embolism or thrombosis of the basilar artery [7]. The second most common cause of LIS is hemorrhage or head trauma [8]. The diagnosis of LIS requires a combination of physical examination abnormalities and confirmatory findings based on neuroimaging using magnetic resonance imaging (MRI) with diffusion-weighted imaging or magnetic resonance angiography (MRA) [9].

Although cocaine use is a well-documented contributing factor in both ischemic and hemorrhagic strokes [10, 11], few cases have been reported in the medical literature describing cocaine as a potential cause of LIS [12]. The mechanism by which cocaine can induce a stroke is not entirely understood. In general, cocaine is thought to work at the presynaptic junction of sympathetic neurons to inhibit the reuptake of noradrenaline, dopamine, and 5-hydroxytryptamine. Cocaine-induced strokes are ischemic in nature, likely due to vasospasm of the cerebral arteries caused by excess neurotransmitters at the synaptic cleft [13]. In addition, cocaine may promote thromboemboli through enhanced platelet aggregation and potentiated endothelial dysfunction [13].

In this case report, we expand the literature and describe an extension of the time frame for onset of cocaine-induced LIS symptoms. Additionally, we review updated reports and proposed pathophysiology of cocaine-induced LIS.

## Case presentation

A 51-year-old African American woman with a limited medical history significant for depression and polysubstance abuse was brought to our emergency department by her boyfriend because of her bizarre behavior, nonsensical communication, and listlessness. As per her boyfriend, the patient was seen inhaling cocaine on the night prior to admission to the hospital. Owing to her altered mental status, her history was obtained from her boyfriend and sister and was limited because of their limited knowledge of the patient’s previous health issues. However, upon further questioning, it was discovered that the patient’s family history was significant for maternal chronic kidney disease, diabetes mellitus, and hypertension. Furthermore, it was revealed that the patient was unemployed, used tobacco, had substance abuse issues specifically with cocaine and heroin, and had abstained from alcohol use for over 10 years. The patient’s initial vital signs were significant for a body temperature of 36.7 °C, heart rate of 60–70 beats/minute, blood pressure of 113/73 mmHg, and respiratory rate of 20 breaths/minute. Her initial physical examination revealed that she was an awake and alert obese African American woman appearing to be her stated age, was confused and very agitated, and was not following commands. She had intact corneal and pupillary reflexes and clear lung sounds. She had regular heart sounds with no murmurs, rubs, or gallops and had a soft nontender abdomen. Additionally, the result of her neurological examination was negative for muscle rigidity, nystagmus, or diffuse hyperhidrosis. However, her examination revealed generalized confusion and agitation. Her pupils were round but sluggishly reactive to light bilaterally. She opened her eyes upon command and moved all four extremities spontaneously, withdrawing appropriately to noxious stimuli, and she exhibited dysarthria.

The result of her urine toxicology was positive for cocaine, opiates, and benzodiazepines. The results of her laboratory studies were unremarkable, with the exception of a potassium level of 3.1 mEq/L. After blood cultures were drawn, empirical intravenous vancomycin and cefepime were initiated for possible encephalomeningitis. The findings of initial unenhanced computed tomography (CT) of the brain were negative for any acute changes. Etomidate and rocuronium were administered for the patient’s agitation, and she was intubated and started on a propofol infusion.

**Fig. 1:**
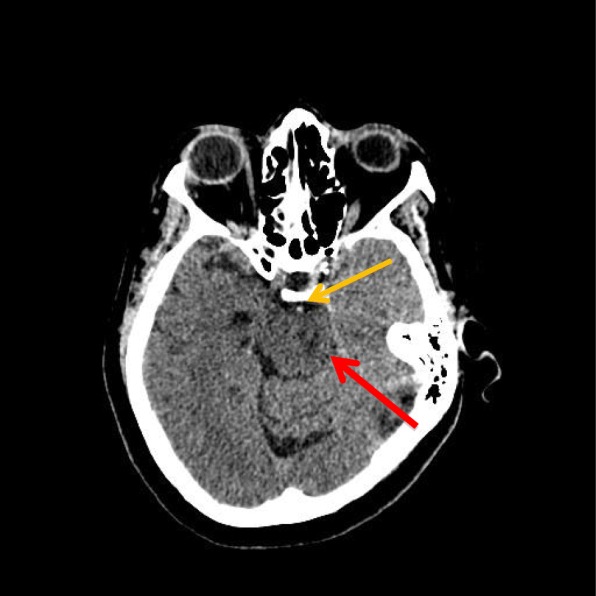
Axial computed tomography of the patient’s head without contrast from February 7, 2015. Image at the level of the brainstem demonstrates a hyperdense basilar artery (*orange arrow*), which in the appropriate clinical setting is concerning for occlusion of the basilar artery due to thrombosis. Also, note that the pons (*red arrow*) is slightly decreased in attenuation compared with the surrounding brain parenchyma, concerning for ischemia. This finding is slightly more prominent on the left side

**Fig. 2:**
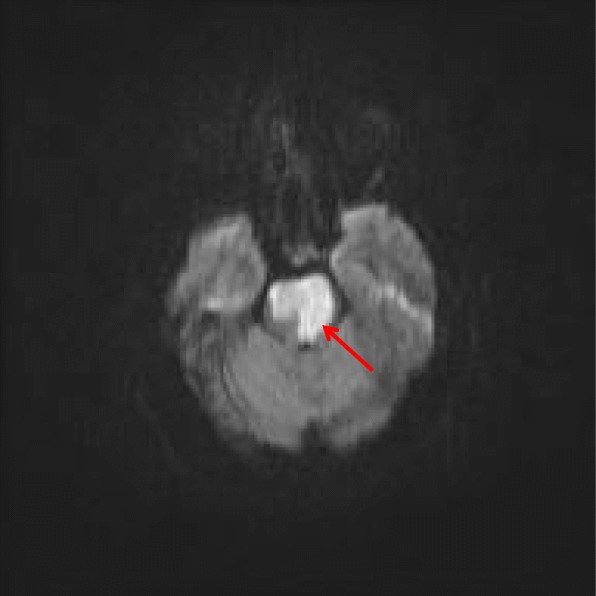
Magnetic resonance imaging of the patient’s brain without contrast from February 9, 2015. A large focus of abnormal restricted diffusion is demonstrated in the brainstem (*red arrow*), consistent with recent infarction. This primarily affects the cranial aspect of the pons, possibly extending into the midbrain. This is a bilateral finding, with more extensive involvement on the left. This is concordant with findings from computed tomography of the patient’s head on February 7, 2015. Mild associated mass effect and swelling are present at this time

**Fig. 3:**
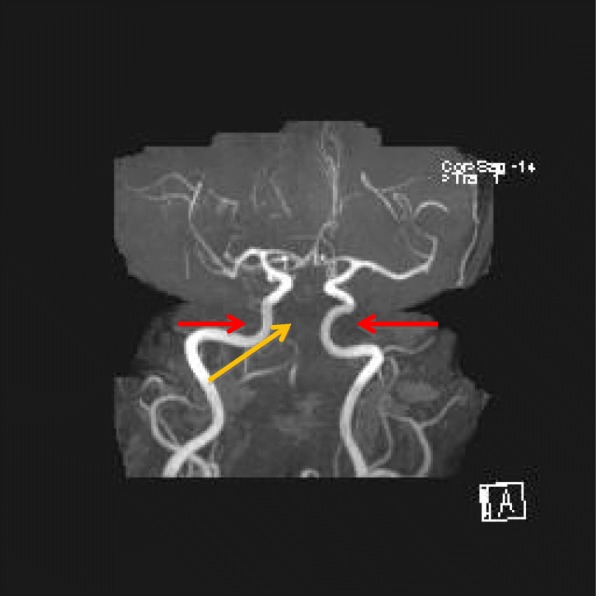
Magnetic resonance angiogram without contrast from February 9, 2015. Complete loss of flow-related signal is noted within the distal basilar artery (*orange arrow*). In contrast, bilateral symmetric flow-related signal in normal-appearing right and left internal cerebral arteries (*red arrows*) is seen

On day 3 of admission, when the patient had been weaned off sedation and paralytic therapy, she demonstrated decorticate posturing, intact gag and cough reflex, positive Babinski reflexes bilaterally, and intact pupillary reflexes. The patient’s motor strength was dramatically decreased in both upper and lower extremities. She was obtunded, unable to follow commands, and opened her eyes spontaneously. On day 4 of admission, she was minimally responsive to commands and was able to communicate only with vertical eye movements. Repeat noncontrast CT showed hyperdensity in the basilar artery, suggestive of occlusion due to thrombosis, which was not present in the initial CT findings (Fig. 1). Findings of lumbar puncture with cerebrospinal fluid examination and electroencephalography (EEG) were unremarkable. MRI showed large foci of abnormally restricted vessels of the brainstem with recent infarction, occurring bilaterally with no observed hemorrhage (Fig. 2). MRA revealed complete loss of flow-related signal within the distal basilar artery (Fig. 3).

Tracheostomy and a percutaneous gastrostomy tube were placed, and supportive care with rehabilitation was initiated. The patient died 2 weeks later of suspected aspiration; however, no autopsy was performed, because her family declined the option.

## Discussion

In this report, we present a case of a 51-year-old woman with a history cocaine abuse who presented to the emergency department for acute behavior changes and was found to have basilar artery thrombosis. Given that few case reports in the literature have described basilar artery thrombosis due to cocaine use, the definitive diagnosis of basilar artery occlusion due to cocaine use may be challenging. Specifically, delayed basilar artery thrombosis due to cocaine use has not been reported previously in the literature. In our patient’s case, we report delayed onset of symptoms and imaging confirmation of basilar artery thrombosis on day 4 of hospitalization.

LIS is a rare neurological disorder characterized by relatively intact cognitive function with preserved awareness and consciousness, but with quadriplegia and inability to speak. According to Smith and Delargy, there are three clinical subcategories of LIS [7]. A patient with classic LIS has quadriplegia and anarthria with preserved consciousness and vertical eye movements. Incomplete LIS is similar to the classic form, but voluntary movements can supplement the presence of vertical eye movements. Total LIS describes a patient who is unable to communicate and has total immobility but retains full consciousness. Our patient’s presentation is consistent with classic LIS because she was able to communicate with vertical eye movements but was fully immobile. She was awake and alert, and her pupils were equally round and reactive to light. Though her corneal reflexes were intact and her vertical eye movement was preserved, she lacked any horizontal eye movements. She had facial plegia and the inability to move any extremities. Furthermore, her lower limbs partially withdrew in response to plantar stimulation. She exhibited bilateral positive Babinski reflexes and absent patellar and Achilles reflexes throughout.

Patients with LIS may be able to communicate with others by moving their eyes or sending blinking messages. Depending on the level of the lesion, vertical eye and upper eyelid movements may not be affected [6]. Furthermore, some patients with incomplete LIS can retain peripheral sensation and proprioception, move certain facial muscles, or control some or all of the extraocular muscles. Aphonia results, due not to the paralysis of the vocal cords but rather to the lack of coordination between breathing and voice, which prevents the voluntary production of sound [14].

Injury to the pons is often due to tissue loss resulting from lack of blood flow, usually from an infarct due to thrombosis or stroke. Other causes of LIS include hemorrhage, infection, loss of myelin, tumors, and certain disorders such as polymyositis or amyotrophic lateral sclerosis [4, 8]. However, due to limited literature, use of cocaine is often not included in the differential diagnosis of LIS.

LIS can be difficult to diagnose because it may mimic loss of consciousness or loss of respiratory control. Recommended brain imaging tests include MRI with close attention to the pons and MRA for thrombosis in the arteries of the brainstem, which can help confirm the diagnosis of LIS in the appropriate clinical scenario and to rule out injuries elsewhere in the brain. Furthermore, an EEG can demonstrate sleep-wake patterns consistent with the apparent inability to physically move [15]. Because these patients lack the ability to execute standard motor responses, such as withdrawal from pain, testing often is conducted through the communication of the patient by blinking or vertical eye movement, which was demonstrated by our patient.

In cases of confirmed cocaine-induced thrombotic or thromboembolic stroke, the main aim is to establish reperfusion therapy within the standard window of time. However, in cases involving posterior cerebral circulation outside the standard time window, intra-arterial thrombolysis and mechanical retrieval thrombectomy may be suggested. Vallée *et al.* and MacEwen e*t al*. described successful thrombosis identification and intra-arterial thrombolysis and aspiration within 30 hours of symptom onset in patients with basilar artery thrombosis induced by cocaine [16, 17]. In both case reports, the patients were young, had no reported significant medical history, and were not comatose at initial presentation. In such patients, intra-arterial thrombolysis with or without mechanical thrombectomy can be considered as a therapeutic option. However, MRI diffusion-weighted and perfusion-weighted imaging should be performed prior to thrombolysis or thrombectomy in order to determine ischemic penumbra and identify salvageable tissue [18]. As reported by Alqahtani *et al.* [12], no endovascular attempt was performed in a 75-year-old man with LIS, owing to the size of the stroke, hemorrhagic conversation, and poor neurological status. As in our patient’s case, the patient’s negative initial findings on imagining combined with late presentation of irreversible neurological symptoms suggested that further interventions would be unsuccessful.

## Conclusion

We suggest that the usual clinical manifestations of LIS can result from cocaine use and, importantly, that cocaine use should be included among the potential causes of LIS. This case report illustrates the need for routine investigation by obtaining a urine toxicology screen for all patients with altered mental status and the importance of awareness of the serious complications of cocaine use.

## Data Availability

Not applicable.

## References

[1] Substance Abuse Mental Health Services Administration (SAMHSA). Results from the 2007 National Survey on Drug Use and Health: National Findings. Office of Applied Studies, NSDUH Series H-34, DHHS Publication No. SMA 08-4343. Rockville: SAMHSA, Office of Applied Studies; 2008. p. 15–29.

[2] Substance Abuse Mental Health Services Administration (SAMHSA). Results from the 2012 National Survey on Drug Use and Health: Summary of National Findings. NSDUH Series H-46, HHS Publication No. (SMA) 13-4795. Rockville: SAMHSA, Office of Applied Studies; 2013. p. 15–29.

[3] León-Carrión J, van Eeckhout P, del Rosario Domínguez-Morales M, Pérez-Santamaría FJ. The locked-in syndrome: a syndrome looking for a therapy. Brain Inj. 2002;16(7):571–82. 10.1080/02699050110119781 12119076

[4] Al-Sardar H, Grabau W. Locked-in syndrome caused by basilar artery ectasia. Br Geriatr Soc. 2002;31:481–2. 10.1093/ageing/31.6.481 12446297

[5] Cairns H, Oldfield RC, Pennybacker JB, *et al.* Akinetic mutism with an epidermoid cyst of the third ventricle. Brain. 1941;64:273–90.

[6] Plum F, Posner J. The diagnosis of stupor and coma. 3rd ed. Philadelphia: F.A. Davis; 1980.

[7] Smith E, Delargy M. Locked-in syndrome. BMJ. 2005;330(7488):406–9. 10.1136/bmj.330.7488.406 PMC549115 15718541

[8] Wyllie AH, Kerr JFR, Currie AR. Cell death: the significance of apoptosis. In: Bourne GH, Danielli JF, Jeon KW, editors. International review of cytology. London: Academic; 1980. p. 251–306. 10.1016/s0074-7696(08)62312-8 7014501

[9] Kotchoubey B, Lotze M. Instrumental methods in the diagnostics of locked-in syndrome. Restor Neurol Neurosci. 2013;31(1):25–40. 10.3233/RNN-120249 23168499

[10] Yoon SH, Zuccarello M, Rapoport RM. Acute cocaine induces endothelin-1-dependent constriction of rabbit basilar artery. Endothelium. 2007;14:137–9. 10.1080/10623320701421644 17578707

[11] Su J, Li J, Li W, Altura BT, Altura BM. Cocaine induces apoptosis in cerebral vascular muscle cells: potential roles in strokes and brain damage. Eur J Pharmacol. 2003;482:61–6. 10.1016/j.ejphar.2003.09.056 14660005

[12] Alqahtani SA, Burger K, Potolicchio S. Cocaine-induced acute fatal basilar artery thrombosis: report of a case and review of the literature. Am J Case Rep. 2015;16:393–7. 10.12659/AJCR.894565 PMC4485643 26109011

[13] Treadwell SD, Robinson TG. Cocaine use and stroke. Postgrad Med J. 2007;83:389–94. 10.1136/pgmj.2006.055970 PMC2600058 17551070

[14] Fager S, Beukelman D, Karantounis R, Jakobs T. Use of safe-laser access technology to increase head movements in persons with severe motor impairments: a series of case reports. Augment Altern Commun. 2006;22(3):222–9. 10.1080/07434610600650318 17114165

[15] Jacome DE, Morilla-Pastor D. Unreactive EEG: pattern in locked-in syndrome. Clin Electroencephalogr. 1990;21(1):31–6. 10.1177/155005949002100112 2297946

[16] MacEwen C, Ward M, Buchan A. A case of cocaine-induced basilar artery thrombosis. Nat Clin Pract Neurol. 2008;4(11):622–6. 10.1038/ncpneuro0879 18679392

[17] Vallée JN, Crozier S, Guillevin R, *et al.* Acute basilar artery occlusion treated by thromboaspiration in a cocaine and ecstasy abuser. Neurology. 2003;61:839–41. 10.1212/wnl.61.6.839 14504335

[18] Albers GW. Expanding the window for thrombolytic therapy in acute stroke: the potential role of acute MRI for patient selection. Stroke. 1999;30:2230–7. 10.1161/01.str.30.10.2230 10512933

